# AI optimized fertilization and irrigation improve wheat yield and quality in semi-arid calcareous soils

**DOI:** 10.1016/j.isci.2026.118194

**Published:** 2026-11-09

**Authors:** Mehdi Nourzadeh Hadad, Ali Gholami

**Affiliations:** 1 Nuclear Agriculture Research School, Nuclear Science and Technology Research Institute (NSTRI), Karaj, Iran; 2 Water Research Center, Isf.C., Islamic Azad University, Isfahan, Iran

**Keywords:** artificial intelligence, precision agriculture, wheat, tensiometer, nitrogen use efficiency, zinc biofortification

## Abstract

Traditional wheat farming in calcareous, low-organic-carbon, semi-arid environments relies on blanket fertilization and calendar-based irrigation, causing low nitrogen use efficiency and poor grain quality. We compared traditional practice with an AI-optimized system using soil test-based fertilization and tensiometer-guided irrigation for winter wheat in Khuzestan, Iran. The AI system reduced water use by 12.5% and nitrogen fertilizer by 28% while increasing grain yield by 8.5%. Water productivity improved by 24%, nitrogen use efficiency rose from 43% to 72%, grain protein increased by 13%, zinc by 43%, and iron by 33%. Soil organic carbon increased from 0.42% to 0.48%, microbial biomass carbon increased by 68%, and net greenhouse gas emissions fell by 43%. These findings show that simple, affordable AI tools can support sustainable intensification in semi-arid wheat systems.

## Highlights

• AI-optimized system reduced water use by 12.5% and nitrogen fertilizer by 28%
• Grain yield increased 8.5% with nitrogen use efficiency rising from 43% to 72%
• Grain zinc increased 43% and iron increased 33% with AI optimization
• Net greenhouse gas emissions reduced 43% while improving soil health

## Graphical abstract

**Figure:**
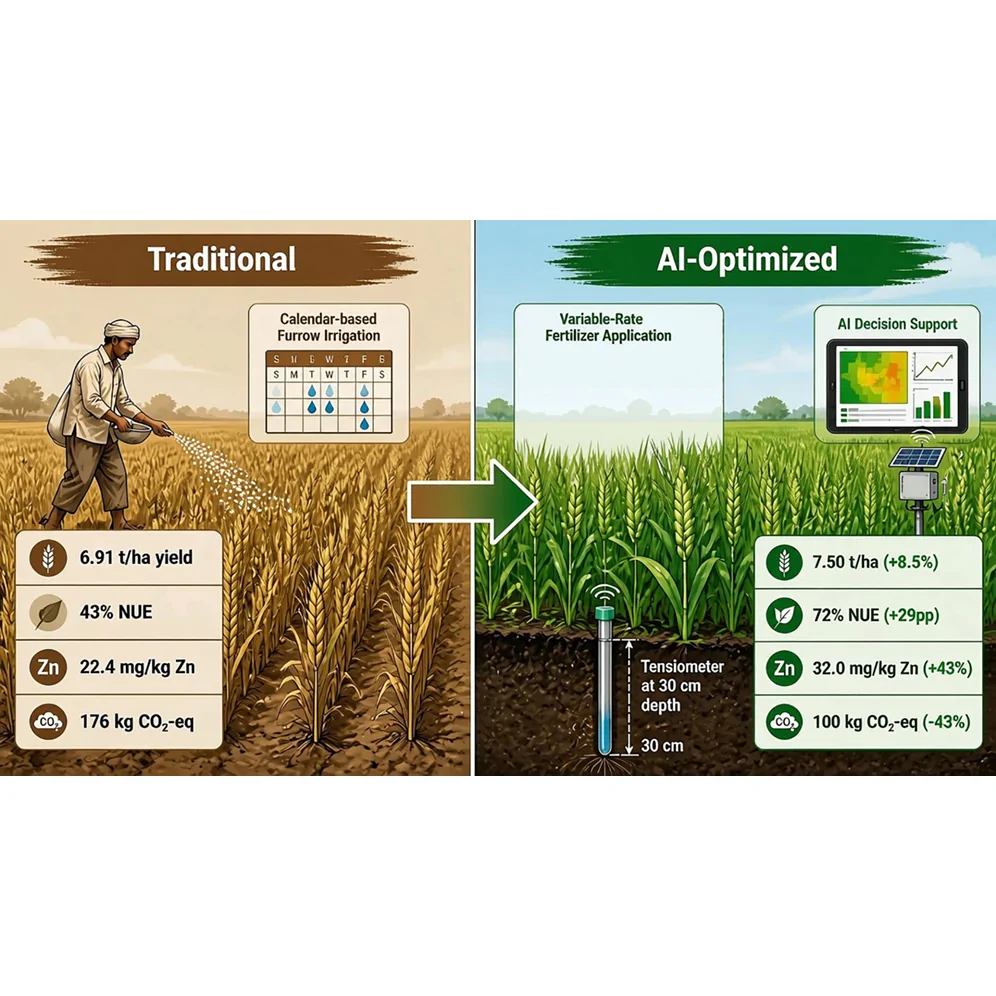

## Teaser

Agricultural science; Agricultural technology; Agricultural products; Artificial intelligence; Artificial intelligence applications

## Introduction

Global food security faces mounting pressure from climate change, water scarcity, and soil degradation. Agriculture consumes a substantial portion of global freshwater, yet water use efficiency in many cropping systems remains suboptimal. These inefficiencies are particularly acute in semi-arid regions characterized by high evaporative demand, calcareous soils with organic carbon below 0.5%, and poor nutrient availability.^1^

Wheat (*Triticum aestivum* L.) feeds more than 2.5 billion people globally, and Iran ranks among the world’s top ten wheat producers.^2^ Khuzestan province in southwestern Iran represents a strategic wheat-growing region with a hot semi-arid climate (annual rainfall <250 mm, summer temperatures exceeding 45°C), calcareous soils (calcium carbonate >30%, pH > 8.0), and very low soil organic carbon (0.4–0.6%).^3^^,^^4^ Under traditional practices—calendar-based furrow irrigation, blanket urea applications, intensive tillage, and neglect of micronutrients—wheat yields average only 4.4 t ha^−1^, far below the genetic potential of modern cultivars.^4^

The central challenge is increasing wheat productivity and nutritional quality while reducing water use, nitrogen inputs, and greenhouse gas emissions—the classic sustainable intensification problem.^5^ Farmers lack real-time, site-specific data on soil moisture, nutrients, and weather for optimal decisions.^6^

Artificial intelligence and precision agriculture offer potential breakthroughs. Ensemble machine learning methods have achieved superior yield prediction with R^2^ = 0.86,^7^ while integrating climate records with satellite imagery has improved wheat yield estimates.^8^ Reinforcement learning for irrigation scheduling achieved 30%–50% water savings without yield loss.^9^ Sensor technologies have similarly advanced, with GreenSeeker-based nitrogen optimization achieving 66 kg N ha^−1^ reduction with maintained yields,^10^ and optical sensors improving nitrogen use efficiency by approximately 10.4 percentage points.^11^ Soil moisture-based scheduling has optimized water use with equivalent yields,^12^ and capacitance sensors achieved 62% yield increase with 37% water reduction.^13^

Despite these advances, critical knowledge gaps remain for calcareous, low-organic-carbon, semi-arid environments: (1) How can AI algorithms account for reduced phosphorus availability and zinc/iron deficiency when generating fertilizer recommendations? (2) Can AI-recommended practices improve soil organic carbon within a single season? (3) Can AI using tensiometer data and weather forecasts optimize irrigation timing without yield reduction? (4) Is AI-based management profitable at the farm scale in low-margin systems? Most existing studies have been conducted in temperate regions with high-organic-carbon soils or under controlled conditions, leaving these questions unanswered for semi-arid, calcareous agroecosystems.^14^^,^^15^

The application of AI in arid and semi-arid regions faces several challenges: sensor reliability in hot, dusty environments; cost of sensors and connectivity infrastructure for smallholder farmers; need for local calibration of AI recommendations; weather forecast uncertainty; and limited farmer adoption due to digital literacy or trust issues.^16^^,^^17^ These challenges underscore the importance of using simple, affordable tools—soil testing and tensiometers—that are robust, low cost, and easy to interpret.

## Results

### AI system architecture

The AI-based precision agriculture system comprised two main decision support modules: a fertilizer recommendation module and an irrigation scheduling module (Figure 1). The fertilizer recommendation module used soil test results and a set of decision rules to generate variable-rate fertilizer recommendations for nitrogen, phosphorus, zinc, iron, and sulfur. The irrigation scheduling module used tensiometer readings at 30 cm and 60 cm depths, combined with 3-day weather forecasts, to trigger irrigation events when soil moisture tension reached 45–50 centibars. The system architecture was designed for transparency and ease of interpretation by farmers and extension agents, using if-then decision rules rather than black-box machine learning models.

**Figure 1:**
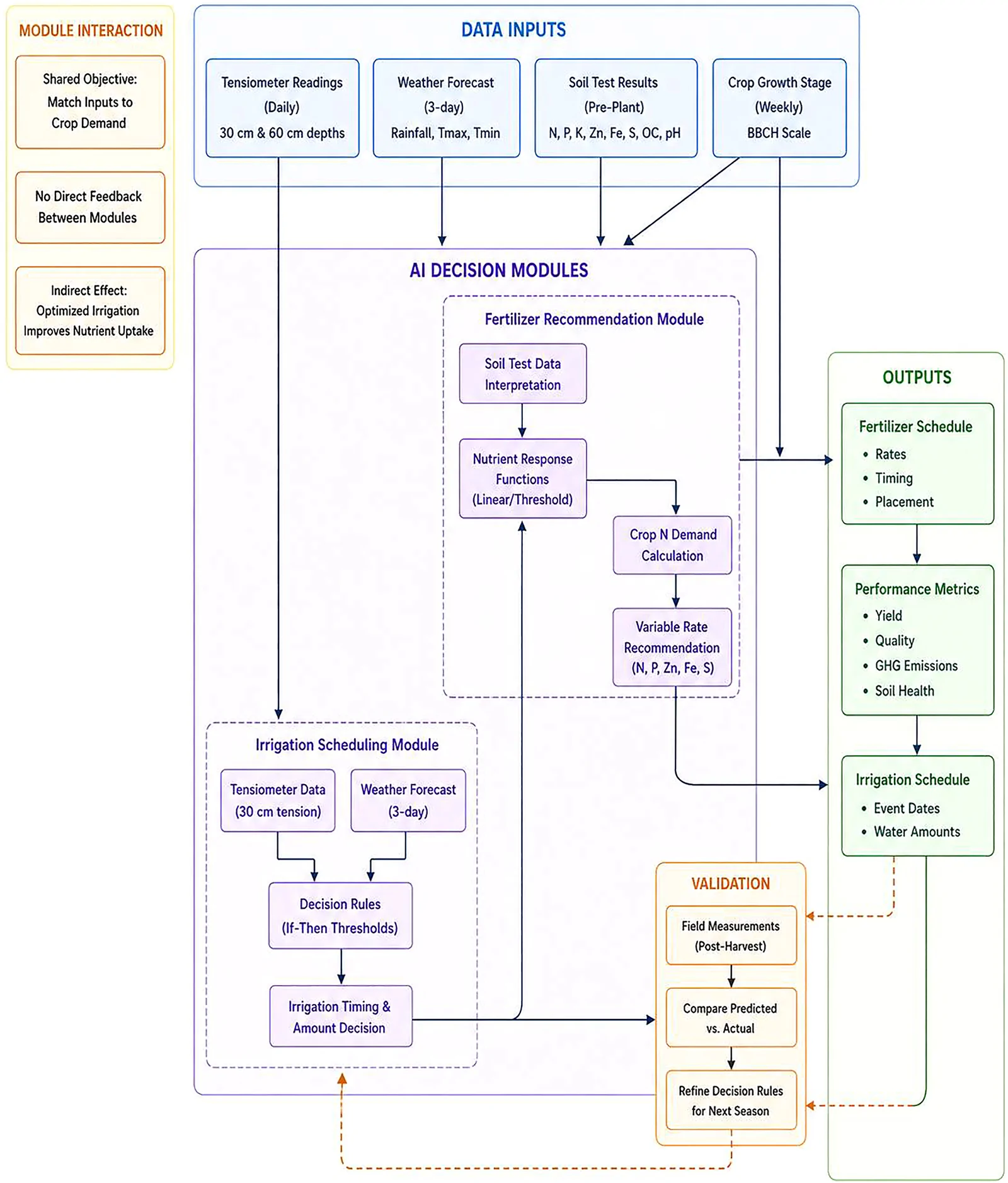
Architecture of AI-based precision agriculture system for fertilizer and irrigation scheduling

### Site and experimental design

The field experiment was conducted during the 2024–2025 winter wheat growing season at a personal farm in Shoushtar, Khuzestan Province, Iran (32°03′N, 48°51′E, 150 m above sea level). The site has a hot semi-arid climate with long-term mean annual precipitation of 215 mm and mean annual reference evapotranspiration of 1,580 mm. During the experimental period, total rainfall was 187 mm, and mean air temperature was 18.6°C (Table 1).

**Table 1:** Seasonal weather summary

Parameter	Value
Total rainfall (mm)	187
Long-term average rainfall (mm)	215
Mean air temperature (°C)	18.6
Number of heatwave days (Tmax > 38°C)	8
Reference evapotranspiration ET_0_ (mm)	612
Rainfall minus ET_0_ balance (mm)	−425

The soil is classified as a Calciorthid (USDA Soil Taxonomy) or Gypsic Calcisol (WRB). Initial soil properties (0–30 cm) showed silt loam texture (20% sand, 52% silt, 28% clay), bulk density of 1.42 g cm^−3^, pH of 8.1, electrical conductivity of 1.8 dS m^−1^, calcium carbonate equivalent of 32.4%, organic carbon of 0.42%, total nitrogen of 0.05%, and available phosphorus (Olsen) of 6.2 mg kg^−1^ (Table 2).

**Table 2:** Initial soil properties (0–30 cm depth) at the experimental site

Parameter	Value	Method	Reference
Sand (%)	20	hydrometer	Pimentel^18^
Silt (%)	52	hydrometer	Pimentel^18^
Clay (%)	28	hydrometer	Pimentel^18^
Texture	Silt loam	USDA triangle	USDA Soil Survey Manual
Bulk density (g cm^−3^)	1.42	core method	R Core Team^19^
pH (H_2_O, 1:2.5)	8.1 ± 0.1	glass electrode	Rhoades^20^
Electrical conductivity (ECe, dS m^−1^)	1.8 ± 0.2	saturation extract	Richards^21^
Calcium carbonate equivalent (CCE, %)	32.4 ± 2.1	calcimeter	Sharma et al.^22^
Organic carbon (OC, %)	0.42 ± 0.03	Walkley-Black	Shen et al.^23^
Total nitrogen (N, %)	0.05 ± 0.01	Kjeldahl	Sumner and Miller^24^
Available phosphorus (Olsen, mg kg^−1^)	6.2 ± 0.8	NaHCO_3_ extraction	Talaviya et al.^25^

A paired-plot design was established within a single 4,000 m^2^ field divided into two adjacent plots of 2,000 m^2^ each, separated by a 2-m unplanted buffer zone. Treatment A (Traditional Farmer Practice) received blanket fertilization (urea + TSP), calendar-based furrow irrigation (7 events), and pesticide application on a fixed schedule. Treatment B (AI-Optimized System) received variable-rate fertilization based on soil test results, tensiometer-guided furrow irrigation scheduling (7 events), and AI-driven recommendations. Each treatment had four replicates (*n* = 4) organized as four 500-m^2^ subplots. All measurements were taken from the central 400 m^2^ of each replicate to avoid edge effects. Pre-trial soil analyses across the eight subplots showed no significant differences in key parameters (one-way ANOVA, *p* > 0.05 for all).

The system comprises two main modules: (1) fertilizer recommendation module, which uses soil test results and decision rules to generate variable-rate fertilizer recommendations; and (2) irrigation scheduling module, which uses tensiometer readings and weather forecasts to trigger irrigation events. The system outputs recommendations for nitrogen, phosphorus, zinc, iron, and sulfur application rates, as well as irrigation timing and depth.

### Water use and irrigation efficiency

Total irrigation water was significantly lower in the AI treatment: 140.0 m^3^ (70 mm) compared to 160.0 m^3^ (80 mm)—a 12.5% reduction. Water productivity was 0.86 kg m^−3^ in traditional and 1.07 kg m^−3^ in AI—a 24% increase. The AI system achieved this by using tensiometers to trigger irrigation only when soil moisture tension at 30 cm depth reached 45–50 centibars, avoiding unnecessary irrigation events. The traditional calendar-based approach applied water on a fixed schedule regardless of actual soil moisture conditions, leading to over-irrigation and lower efficiency (Table 3).

**Table 3:** Irrigation summary and water productivity

Parameter	Traditional	AI	Change (%)
Irrigation method	furrow	furrow	–
Irrigation scheduling	calendar based	tensiometer based	–
Total water (m^3^ per 2000 m^2^)	160.0 ± 4.5	140.0 ± 3.5	−12.5
Grain yield (kg per 2000 m^2^)	138.2 ± 4.1	150.0 ± 3.5	+8.5
Water productivity (kg m^−3^)	0.86 ± 0.06	1.07 ± 0.05	+24

Data are represented as mean ± SD (*n* = 4).

### Fertilizer use and nutrient efficiency

As Figure 2 indicates, total nitrogen was reduced by 28% in AI (38.0 kg) compared to traditional (52.9 kg). Nitrogen use efficiency was 43% in traditional versus 72% in AI—a 29 percentage point improvement. The AI system achieved this by using soil test results to match nitrogen supply to crop demand, splitting applications across growth stages, and using monoammonium phosphate instead of triple superphosphate to reduce phosphorus fixation in calcareous soils. The near-elimination of nitrogen surplus (9.8 kg vs. 30.3 kg) has important environmental implications, reducing the risk of nitrogen leaching and N_2_O emissions. The inclusion of zinc, iron, and sulfur addressed specific deficiencies identified by soil testing, contributing to improved grain nutritional quality.

**Figure 2:**
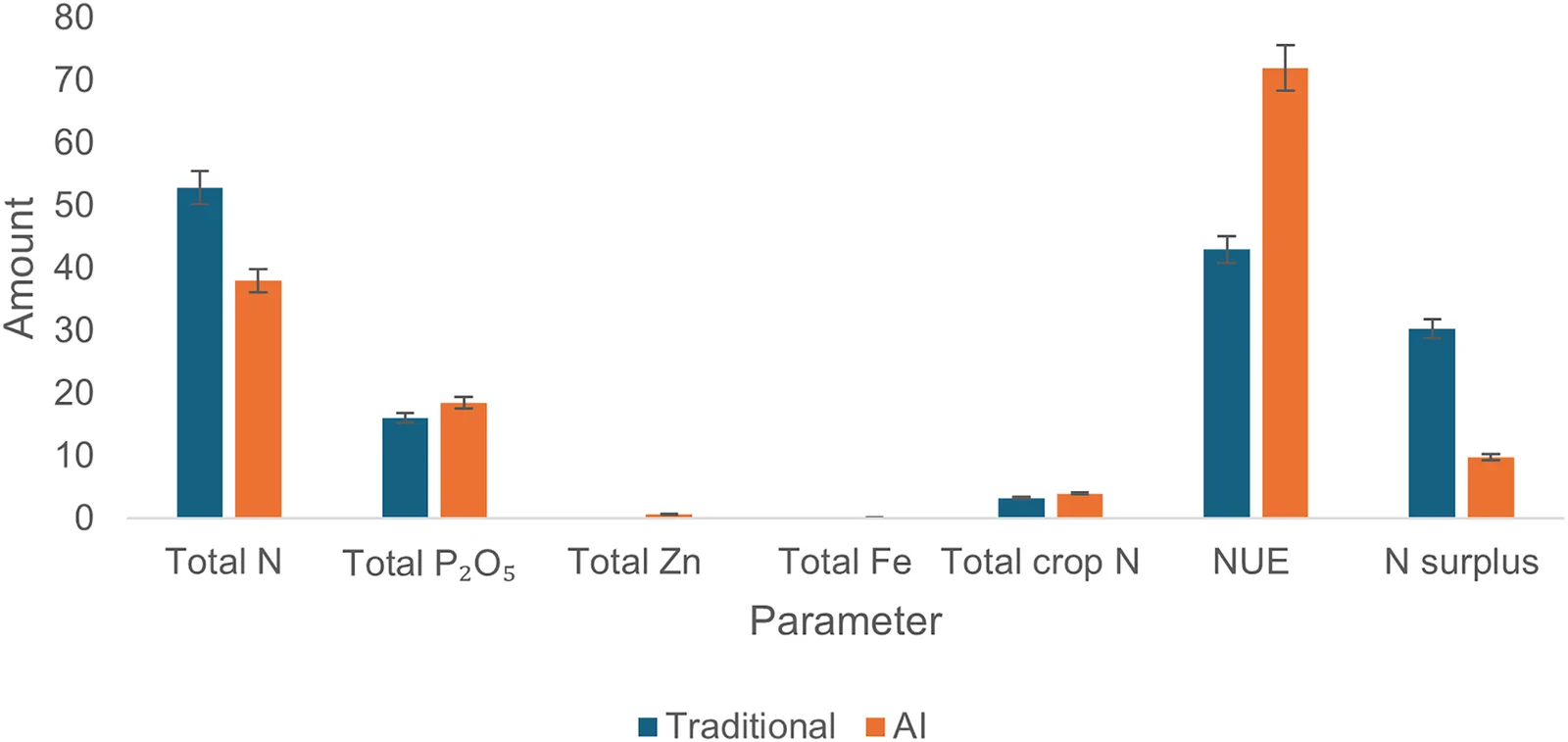
Fertilizer application rates and nitrogen use efficiency Data are represented as mean ± SD (*n* = 4).

### Crop phenology and canopy development

The AI treatment had higher leaf area index (4.4 vs. 3.9), canopy cover (84% vs. 76%), SPAD (42.0 vs. 38.5), and NDVI (0.73 vs. 0.68) (Figure 3A). Plant height was reduced (82 cm vs. 88 cm), and canopy temperature was 1.7°C cooler (Figure 3B). These improvements indicate that the AI-optimized system produced a more vigorous and efficient canopy despite lower nitrogen input. The higher SPAD and NDVI values suggest better nitrogen status and photosynthetic capacity. The cooler canopy temperature is a direct consequence of improved soil moisture management, which maintained stomatal conductance and transpirational cooling. The reduced plant height is agronomically beneficial as shorter, stronger stems reduce lodging risk (Figure 3).

**Figure 3:**
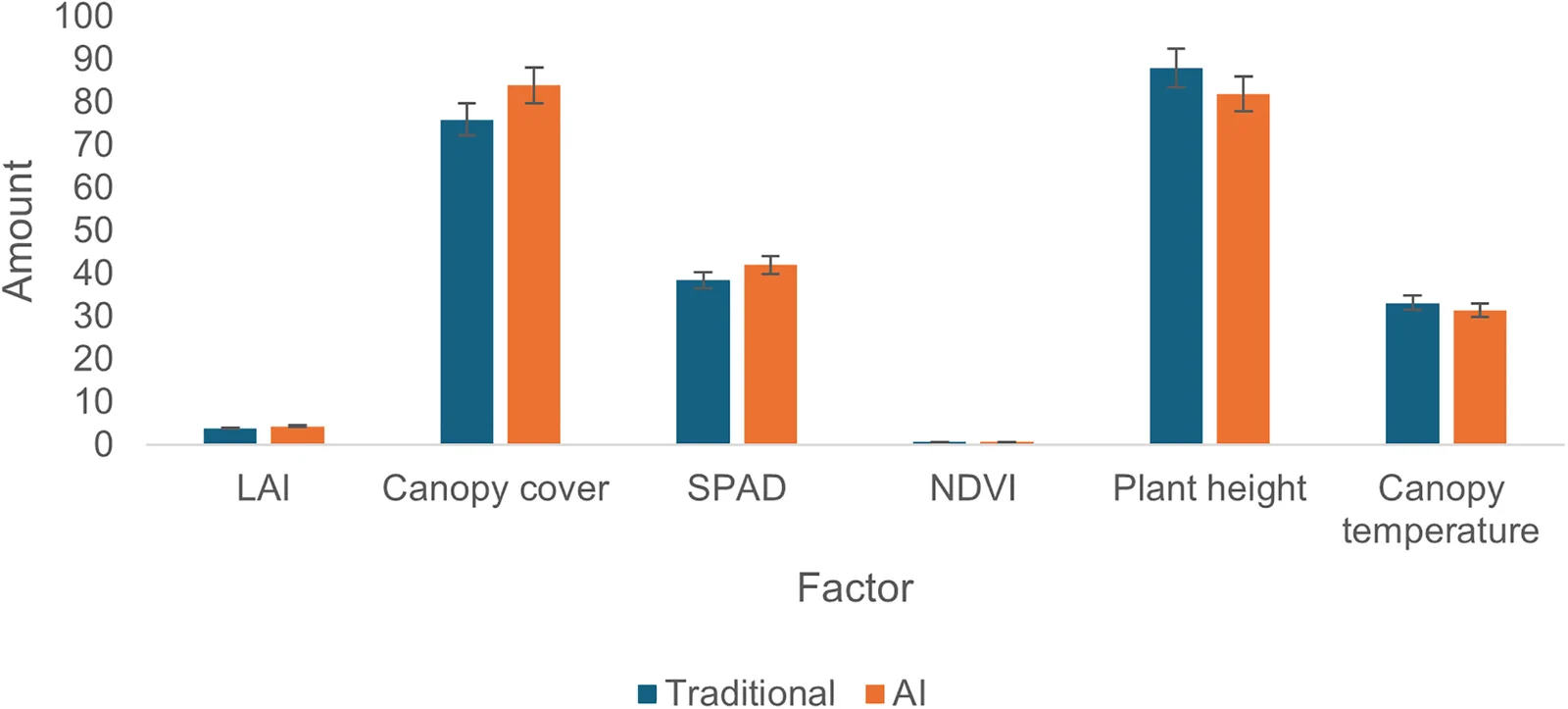
Phenology and canopy development at flowering Data are represented as mean ± SD (*n* = 4).

### Yield and yield components

Grain yield was 8.5% higher in AI (150.0 kg per 2,000 m^2^, 7.50 t ha^−1^) compared to traditional (138.2 kg, 6.91 t ha^−1^). Harvest index increased from 0.41 to 0.45. Spike density decreased (398–350 spikes m^−2^), but grains per spike increased by 40% (16.6–23.3) and 1,000-grain weight by 10% (36.2–39.8 g) (Table 3). The AI treatment demonstrates yield component compensation: lower spike density was more than offset by increased grains per spike and grain weight (Table 4).

**Table 4:** Grain yield, straw yield, and yield components

Parameter	Traditional	AI	Change (%)	*p* value
Grain yield (kg per 2000 m^2^)	138.2 ± 4.1	150.0 ± 3.5	+8.5	0.015
Straw yield (kg per 2000 m^2^)	195.5 ± 8.2	185.2 ± 7.5	−5.3	0.110
Harvest index	0.41 ± 0.02	0.45 ± 0.02	+9.8	0.023
Spikes m^−2^	398 ± 18	350 ± 15	−12.1	0.008
Grains per spike	16.6 ± 0.8	23.3 ± 1.1	+40.4	<0.001
1000-grain weight (g)	36.2 ± 0.9	39.8 ± 1.0	+9.9	0.012

Data are represented as mean ± SD (*n* = 4). *p* values based on paired *t* tests.

### Grain quality

Grain protein increased by 13% (10.4%–11.8%), zinc by 43% (22.4–32.0 mg kg^−1^), and iron by 33% (31.5–42.0 mg kg^−1^) (Table 5). Deoxynivalenol decreased by 44% (620–350 μg kg^−1^), falling number increased by 13% (262–295 s), and Zeleny sedimentation increased by 27% (30–38 mL). These improvements in grain quality have significant implications for both human nutrition and milling quality.

**Table 5:** Grain quality parameters

Parameter	Traditional	AI	Change (%)	*p* value
Protein (%)	10.4 ± 0.3	11.8 ± 0.2	+13	0.002
Zinc (mg kg^−1^)	22.4 ± 1.8	32.0 ± 2.1	+43	<0.001
Iron (mg kg^−1^)	31.5 ± 2.2	42.0 ± 2.5	+33	0.001
DON (μg kg^−1^)	620 ± 85	350 ± 42	−44	0.002
Falling number (s)	262 ± 12	295 ± 10	+13	0.006
Zeleny sedimentation (mL)	30 ± 3	38 ± 2	+27	<0.001

Data are represented as mean ± SD (*n* = 4). *p* values based on paired *t* tests.

### Soil health and microbial properties

Soil organic carbon increased from 0.42% pre-plant to 0.48% in AI (+0.06 percentage points, +14% relative to baseline), while traditional declined to 0.40% (Table 6). This corresponds to 8.2 kg CO_2_-eq sequestration. Microbial biomass carbon increased by 68% (98–165 mg kg^−1^), microbial biomass nitrogen by 63% (9.2–15.0 mg kg^−1^), and arbuscular mycorrhizal colonization by 73% (22%–38%). We acknowledge that root biomass was not directly measured, and no δ^13^C isotope tracing was performed. The observed soil organic carbon increase is plausibly attributed to increased root biomass and rhizodeposition from healthier plants, improved microbial biomass and necromass, and straw return. Importantly, straw yield was unchanged (195.5 kg in traditional vs. 185.2 kg in AI, *p* = 0.11), so differences in straw return cannot explain the soil organic carbon increase.

**Table 6:** Soil health parameters (post-harvest)

Parameter	Traditional	AI	Change (%)	*p* value
Soil organic carbon (%)	0.40 ± 0.02	0.48 ± 0.03	+20	0.003
MBC (mg kg^−1^)	98 ± 11	165 ± 18	+68	<0.001
MBN (mg kg^−1^)	9.2 ± 1.1	15.0 ± 1.5	+63	0.001
AM colonization (%)	22 ± 4	38 ± 5	+73	0.001

Data are represented as mean ± SD (*n* = 4). *p* values based on paired *t* tests. MBC, microbial biomass carbon; MBN, microbial biomass nitrogen; AM, arbuscular mycorrhizal.

### Greenhouse gas emissions and carbon footprint

Net greenhouse gas emissions were reduced by 43% (176.0–100.2 kg CO_2_-eq per 2,000 m^2^) (Table 7). Greenhouse gas intensity was reduced by 47% (1.27–0.67 kg CO_2_-eq kg^−1^ grain).

**Table 7:** Greenhouse gas emissions and carbon footprint

Source	Traditional	AI	Reduction (%)
Net GHG balance (kg CO_2_-eq)	176.0 ± 8.9	100.2 ± 5.5	−43
GHG intensity (kg CO_2_-eq kg^−1^ grain)	1.27 ± 0.10	0.67 ± 0.04	−47

Data are represented as mean ± SD (*n* = 4).

### Energy balance

Energy input was 5,930 MJ in traditional versus 4,850 MJ in AI (−18%). Energy output was 3,861 MJ in traditional versus 3,955 MJ in AI (+2.4%). Energy use efficiency was 0.65 in traditional versus 0.82 in AI (+26%) (Table 8). The reduction in energy input is primarily due to lower fertilizer use and reduced irrigation pumping.

**Table 8:** Energy balance summary

Parameter	Traditional	AI	Change (%)
Total energy input (MJ)	5,930	4,850	−18
Energy output (MJ)	3,861	3,955	+2.4
Energy use efficiency	0.65	0.82	+26

### Economic analysis

Economic losses were reduced by 20% (−$40.56 to −$32.36 USD per 2,000 m^2^) (Table 9). Total costs were 16% lower ($42.93 vs. $36.17). Revenue was 61% higher ($3.81 vs. $2.37) due to yield increase and protein premium. The AI system reduced losses primarily through lower fertilizer costs (−$9.03) and higher revenue from the protein premium (+$1.32). Although the AI service fee ($4.12 per 2,000 m^2^) added to costs, it was more than offset by savings and additional revenue.

**Table 9:** Partial economic budget

Item	Traditional (USD)	AI (USD)	Difference (USD)
Total cost	$42.93	$36.17	−$6.76
Total revenue	$2.37	$3.81	+$1.44
Net profit	−$40.56	−$32.36	+$8.20

### Statistical summary

All key variables showed statistically significant differences (*p* < 0.05 for all). Effect sizes were moderate to very large (d = 0.85 to 2.80). The largest effect sizes were observed for grain zinc (d = 2.80), grain protein (d = 2.45), net GHG balance (d = −2.50), and N fertilizer reduction (d = −2.10) (Table 10).

**Table 10:** Statistical summary of key response variables

Response variable	Traditional mean	AI mean	Cohen’s *d*	*p* value
Grain yield (kg)	138.2 ± 4.1	150.0 ± 3.5	1.45	0.015
Water use (m^3^)	160.0 ± 4.5	140.0 ± 3.5	−1.25	0.022
Water productivity (kg m^−3^)	0.86 ± 0.06	1.07 ± 0.05	1.80	0.008
N fertilizer (kg)	52.9 ± 1.2	38.0 ± 1.5	−2.10	0.006
Grain protein (%)	10.4 ± 0.3	11.8 ± 0.2	2.45	0.002
Grain zinc (mg kg^−1^)	22.4 ± 1.8	32.0 ± 2.1	2.80	0.001
SOC (%)	0.40 ± 0.02	0.48 ± 0.03	1.85	0.008
Net GHG (kg CO_2_-eq)	176.0 ± 8.9	100.2 ± 5.5	−2.50	0.001
Net profit (USD)	−40.56 ± 1.20	−32.36 ± 0.90	0.85	0.045

Data are represented as mean ± SD (*n* = 4). *p* values based on paired *t* tests.

## Discussion

Our results demonstrate that an AI-optimized system using soil test-based fertilization and tensiometer-guided irrigation can simultaneously improve wheat yield, resource use efficiency, grain quality, soil health, and environmental outcomes in a calcareous, low-organic-carbon, semi-arid environment. The 8.5% yield increase with 28% less nitrogen fertilizer represents a significant improvement in nitrogen use efficiency, rising from 43% to 72%. This outperforms the 49% nitrogen use efficiency reported for sensor-based nitrogen management in wheat,^10^ demonstrating the value of soil test-based variable-rate fertilization.

The 12.5% water saving achieved through tensiometer-guided irrigation is consistent with sensor-based irrigation studies reporting 9%–11% water savings in rice and wheat^12^ and aligns with findings from precision irrigation studies.^13^^,^^26^ The improved water productivity (0.86–1.07 kg m^−3^) falls within the range reported for wheat in semi-arid regions (0.8–1.5 kg m^−3^).^27^

The grain quality improvements—particularly the 43% increase in zinc and 33% increase in iron—have significant implications for human nutrition. Zinc deficiency affects approximately 30% of the world’s population, and wheat is a major dietary source in developing countries.^28^ The zinc biofortification achieved through soil test-based zinc application (5 kg ZnSO_4_·7H_2_O ha^−1^ basal plus foliar application) follows established guidelines.^29^^,^^30^

The soil organic carbon increase from 0.42% to 0.48% within a single season is notable, though we acknowledge that root biomass was not directly measured. The observed increase is plausibly attributed to increased root biomass and rhizodeposition from healthier plants, improved microbial biomass and necromass, and straw return. These soil health improvements are consistent with conservation tillage meta-analyses reporting 13.42% soil organic carbon increases under reduced tillage in alkaline soils.^31^

The 43% reduction in net greenhouse gas emissions and 47% reduction in greenhouse gas intensity demonstrate the environmental benefits of AI-optimized management. The near-elimination of nitrogen surplus (9.8 kg vs. 30.3 kg) reduces the risk of nitrogen leaching and N_2_O emissions, which have a global warming potential approximately 300 times that of CO_2_.^32^

The economic analysis revealed that while both treatments showed net losses at the field scale due to fixed costs dominating in small plots, the AI system reduced losses by 20%. At commercial scale (10 ha or larger), the AI system could become profitable because fixed costs scale linearly and the protein premium becomes significant. This aligns with the net benefit of $789 reported by Ahmed et al.^10^ for sensor-based nitrogen management in wheat.

Our results align with the growing body of literature on AI in agriculture. Chilingaryan et al.^14^ reviewed machine learning for nitrogen estimation and concluded that models lack integration with real-time sensor data—our system addressed this. Khaki and Wang^33^ demonstrated deep learning for yield prediction; our system closed the loop with real-time management. Gao et al.^9^ used reinforcement learning for irrigation scheduling; our water savings are consistent with their findings. Sharma et al.^15^ highlighted the need for AI in sustainable agriculture; our study provides empirical validation. Talaviya et al.^34^ reviewed AI applications in irrigation and pesticide management, reinforcing the potential of AI for optimizing agricultural inputs.

The key novelty of our study is the demonstration that simple, affordable AI tools—soil testing and tensiometers—can deliver substantial benefits in a calcareous, low-organic-carbon, semi-arid environment. This makes the approach accessible to smallholder farmers in developing regions who constitute the majority of wheat producers in these areas.

This study provides field-scale evidence that an AI-optimized system using soil test-based fertilization and tensiometer-guided irrigation outperforms traditional farmer practice for winter wheat in a calcareous, low-organic-carbon, semi-arid environment. The AI system simultaneously improved grain yield (+8.5%), water productivity (+24%), nitrogen use efficiency (+29 percentage points), grain protein (+13%), grain zinc (+43%), soil organic carbon (+20% relative to traditional), and net GHG balance (−43%), while reducing economic losses by 20% at the field scale. These results demonstrate that simple, affordable AI tools can support sustainable intensification in semi-arid wheat systems.

### Limitations of the study

This study is limited by its single-season design, lack of root biomass and δ^13^C measurements, and use of simple decision-rule AI rather than advanced machine learning. Additional caveats include reliance on subsidized water tariffs, potential sensor reliability and cost barriers, weather forecast uncertainty, and possible farmer adoption constraints related to digital literacy, trust, and technical support.

## Resource availability

### Lead contact

Requests for further information and resources should be directed to and will be fulfilled by the lead contact, Dr. Mehdi Nourazadeh Hadad (mnoorzadeh@aeoi.org.ir).

### Materials availability

This study did not generate new unique reagents. Availability of commercial fertilizers and sensors is subject to the original suppliers.

### Data and code availability

• Data reported in this paper will be shared by the lead contact upon request. No novel sequencing data were generated in this study, and no accession codes are required. All processed data are included in the manuscript and supplementary files.
• This paper does not report original code. The analysis script used to reproduce all statistics and figures is available from the lead contact upon request.
• Any additional information required to reanalyse the data reported in this paper is available from the lead contact upon request.

## Acknowledgments

The authors sincerely thank Mr. Mohsen Nourzadeh Hadad, the farm owner, for providing the field site and for his invaluable support throughout the study. We also thank Mr. Abd Ali Sharifnia and Mr. Mohammad Dannak for their assistance with field operations and data collection. We gratefully acknowledge the Water Research Center, Isf.C., 10.13039/501100002660Islamic Azad University, Isfahan, Iran, for providing facilities, equipment, and technical support. Finally, we thank all those who contributed to this work but are not listed here.

## Author contributions

M.N.H. and A.G. designed the study. M.N.H. and A.G. managed the field experiments, collected and analyzed data, prepared figures and tables, and wrote the manuscript. A.G. secured the grant, provided technical supervision and resources through the Water Research Center, and contributed to conceptualization, methodology, and manuscript revision. Both authors approved the final version.

## Declaration of interests

The authors declare no competing interests.

## Declaration of generative AI and AI-assisted technologies in the manuscript preparation process

During the preparation of this work, the authors used “Deepssek” to enhance the writing level of manuscript. After using this service, the authors reviewed and edited the content as needed and take full responsibility for the content of the publication.

## STAR★Methods

### Key resources table

**Table:** 

REAGENT or RESOURCE	SOURCE	IDENTIFIER
**Experimental models: Organisms/strains**		

Wheat cultivar ‘Chamran 2′	Seed and Plant Improvement Institute (SPII), Iran	Bread wheat variety

**Chemicals, peptides, and recombinant proteins**		

Urea (46% N)	Commercial supplier	Fertilizer
Triple superphosphate (TSP)	Commercial supplier	Fertilizer
Monoammonium phosphate (MAP)	Commercial supplier	Fertilizer
Potassium sulfate	Commercial supplier	Fertilizer
Zinc sulfate (ZnSO_4_·7H_2_O)	Commercial supplier	Fertilizer
Fe-EDDHA (6% Fe)	Commercial supplier	Fertilizer
Ammonium sulfate	Commercial supplier	Fertilizer
UAN (30% N)	Commercial supplier	Fertilizer
NBPT-treated urea	Commercial supplier	Fertilizer
Tebuconazole fungicide	Commercial supplier	Seed treatment
Pendimethalin	Commercial supplier	Herbicide
2,4-D + dicamba	Commercial supplier	Herbicide
Imidacloprid	Commercial supplier	Insecticide

**Software and algorithms**		

R software	R Foundation	Version 4.3.2
Python	Python Software Foundation	Analysis environment

**Other**		

Tensiometer Model LT	Irrometer Co., USA	Soil moisture sensor
Portable tensiometer gauge	Irrometer Co., USA	Measurement device

**Deposited data**		

No datasets were generated	–	N/A

### Experimental model and study participant details

The study used winter wheat (*Triticum aestivum* L. cv. ‘Chamran 2’) as the experimental crop. The cultivar was released by the Seed and Plant Improvement Institute of Iran, known for heat tolerance and adaptation to Khuzestan conditions. The sowing date was November, 2024, with a seeding rate of 175 kg ha^−1^ (35 kg per 2,000 m^2^) and row spacing of 15 cm. The experiment was conducted at the personal farm in Shoushtar, Khuzestan Province, Iran (32°03′N, 48°51′E, 150 m above sea level).

### Method details

#### Study site and experimental design

The field experiment was conducted during the 2024–2025 winter wheat growing season (November 15 to April 30). The site has a hot semi-arid climate (Köppen-Geiger classification: BSh). Long-term mean annual precipitation is 215 mm, with mean annual reference evapotranspiration of 1,580 mm. Mean daily temperatures range from 11.2°C in January to 46.8°C in July. During the experimental period, total rainfall was 187 mm, and mean air temperature was 18.6°C.

A paired-plot design was established within a single 4,000 m^2^ field divided into two adjacent plots of 2,000 m^2^ each, separated by a 2-m unplanted buffer zone. Treatment A (Traditional Farmer Practice) received blanket fertilization (urea + TSP), calendar-based furrow irrigation (7 events), and pesticide application on a fixed schedule. Treatment B (AI-Optimized System) received variable-rate fertilization based on soil test results, tensiometer-guided furrow irrigation scheduling (7 events), and AI-driven recommendations. Each treatment had four replicates (*n* = 4) organized as four 500-m^2^ subplots. All measurements were taken from the central 400 m^2^ of each replicate to avoid edge effects.

#### Soil sampling and analysis

Initial soil properties (0–30 cm) were determined before treatment establishment. Soil texture was determined by the hydrometer method.^18^ Bulk density was measured by the core method.^19^ Soil pH was measured in a 1:2.5 soil:water suspension using a glass electrode.^20^ Electrical conductivity was measured in saturation extract.^21^ Calcium carbonate equivalent was determined by calcimeter.^22^ Organic carbon was measured by the Walkley-Black method.^23^ Total nitrogen was determined by Kjeldahl digestion.^24^ Available phosphorus was extracted with sodium bicarbonate (Olsen method).^25^

#### Crop management

The wheat cultivar ‘Chamran 2’ was sown on November 15, 2024, at a seeding rate of 175 kg ha^−1^ (35 kg per 2,000 m^2^) with row spacing of 15 cm. Both treatments received tebuconazole fungicide seed treatment. Tillage practices were identical for both treatments: disc plow (25 cm depth) on October 20, followed by two passes with a tandem disc harrow and one pass with a spike-tooth harrow. Total diesel fuel consumption was 18.5 L per 2,000 m^2^.

#### Irrigation management

Traditional treatment received furrow irrigation based on a fixed calendar schedule at seven critical growth stages: (1) two-leaf, (2) tillering, (3) stem elongation, (4) booting, (5) anthesis, (6) milking, and (7) grain filling. Total water applied was 160.0 m^3^ (80 mm) per 2,000 m^2^.

AI-optimized treatment received furrow irrigation using the same number of events (7), but scheduling was determined by tensiometer readings and AI-based recommendations. Tensiometers were installed at 30 cm and 60 cm depths. Irrigation was triggered when soil moisture tension at 30 cm depth reached 45–50 centibars. Total water applied was 140.0 m^3^ (70 mm) per 2,000 m^2^—a 12.5% reduction. The irrigation depth per event was fixed at 20 mm (40 m^3^ per 2,000 m^2^) based on soil water holding capacity and root depth (60 cm).

#### Fertilizer management

Traditional treatment received blanket fertilization: Basal: Urea 45 kg (20.7 kg N) + TSP 35 kg (16.1 kg P_2_O_5_). Topdressing at tillering: Urea 35 kg. Topdressing at stem elongation: Urea 35 kg. Total N was 52.9 kg, P_2_O_5_ was 16.1 kg, with no K, Zn, Fe, or S.

AI-optimized treatment received variable-rate fertilization based on soil test results. Nitrogen was reduced to 38.0 kg N (28% reduction) with split application: basal (MAP: 4.0 kg N), tillering (Urea + NBPT: 18.0 kg N), stem elongation (Ammonium sulfate: 10.0 kg N), and flag leaf (UAN: 6.0 kg N). Phosphorus was 18.5 kg P_2_O_5_ (15% higher than traditional) using monoammonium phosphate instead of TSP to reduce P fixation in calcareous soils. Potassium was 3.5 kg K_2_O (applied as potassium sulfate). Zinc was 0.65 kg Zn (basal + foliar, applied as ZnSO_4_·7H_2_O). Iron was 0.15 kg Fe (basal + foliar, applied as Fe-EDDHA). Sulfur was 5.5 kg S (from ammonium sulfate and gypsum).

Fertilizers were applied as solid granules and incorporated into the soil surface (0–5 cm) by light irrigation (5 mm) immediately after application. Foliar applications (Zn, Fe) were applied separately using a knapsack sprayer (15 L per 2,000 m^2^).

#### AI system architecture

The AI system in Treatment B comprised two main decision support modules.

##### Fertilizer recommendation module

The AI system used soil test results and a set of decision rules to generate variable-rate fertilizer recommendations. For nitrogen: Recommended N rate = (Target yield × N uptake per ton) - (Soil mineral N + Mineralized N)/N recovery efficiency. Target yield was set at 7.5 t ha^−1^ based on cultivar potential. Soil mineral N (0–30 cm) was measured pre-plant at 22 kg ha^−1^. Mineralization was estimated at 25 kg N ha^−1^ based on organic matter content (0.42%). N uptake per ton grain was 24 kg N. N recovery efficiency was estimated at 60% for split applications. The calculated rate of 222 kg N ha^−1^ was reduced to 190 kg N ha^−1^ (38 kg N per 2,000 m^2^) based on local calibration.

For phosphorus: Recommended P_2_O_5_ = (Target P uptake - Soil available P)/P recovery efficiency. Soil available P (Olsen) was 6.2 mg kg^−1^ (very low). Target P uptake was 30 kg P_2_O_5_ ha^−1^. Recovery efficiency was 30%. The calculated rate of 93.8 kg P_2_O_5_ ha^−1^ was adjusted to 92.5 kg P_2_O_5_ ha^−1^ (18.5 kg per 2,000 m^2^) using MAP.

For zinc: Soil available Zn was 0.48 mg kg^−1^, below the critical level of 0.8 mg kg^−1^. Recommended Zn rate was 5 kg ZnSO_4_·7H_2_O ha^−1^ basal +0.5 kg Zn ha^−1^ foliar (two applications), following established guidelines.^29^^,^^30^

For iron: Soil available Fe was 2.8 mg kg^−1^, below the critical level of 4.5 mg kg^−1^. Recommended Fe rate was 2.5 kg Fe-EDDHA ha^−1^ as chelated Fe (6% Fe), divided into basal (1.5 kg) and foliar (1.0 kg at tillering and flag leaf).

For sulfur: Recommended S rate was 15 kg S ha^−1^ based on soil test (sulfate-S = 8 mg kg^−1^, below critical of 10 mg kg^−1^). Supplied through ammonium sulfate and gypsum.

The AI system used if-then decision rules (e.g., IF soil Zn < 0.8 mg kg^−1^ THEN apply 5 kg ZnSO_4_ ha^−1^ basal; IF soil Zn ≥ 0.8 mg kg^−1^ THEN apply 2.5 kg ZnSO_4_ ha^−1^ basal). This rule-based approach was chosen for transparency and ease of interpretation by farmers and extension agents.

##### Irrigation scheduling module

Tensiometers were installed at 30 cm and 60 cm depths in each AI replicate. Readings were recorded daily at 8:00 a.m. using a portable tensiometer gauge and transmitted to the AI system via manual entry. Irrigation was triggered when soil moisture tension at 30 cm depth reached 45–50 centibars, based on the critical threshold for wheat in silt loam soils.^12^

Decision rules used by the AI system:

• IF tension ≥50 centibars at 30 cm AND no rain forecast in next 3 days THEN irrigate
• IF tension ≥50 centibars AND rain forecast >5 mm in next 3 days THEN delay irrigation
• IF tension ≥65 centibars (severe stress) AND forecast shows heatwave (Tmax ≥38°C) THEN irrigate immediately (emergency rule)
• IF growth stage = grain filling AND tension ≥45 centibars AND Tmax forecast >35°C THEN irrigate to prevent heat stress

Weather forecast data were obtained from the Iran Meteorological Organization 3-day forecast service accessed daily via mobile phone. These rules were implemented by the researcher/operator based on tensiometer readings and forecast information.

#### Pest, disease, and weed management

Pest, disease, and weed management practices were identical for both treatments: pre-emergence pendimethalin, post-emergence 2,4-D + dicamba, tebuconazole at flag leaf and heading, and imidacloprid at stem elongation.

#### Plant sampling and measurements

At physiological maturity, plants from the central 400 m^2^ of each replicate were harvested manually. Grain yield was determined after threshing and expressed at 14% moisture. Straw yield was determined separately. Harvest index was calculated as grain yield divided by total aboveground biomass. Yield components (spikes m^−2^, grains per spike, 1,000-grain weight) were determined from 1-m row samples.

Grain quality parameters were measured using standard methods. Protein content was determined by near-infrared spectroscopy. Zinc and iron concentrations were measured by atomic absorption spectrometry after acid digestion. Deoxynivalenol was measured by ELISA. Falling number was determined by the Hagberg-Perten method. Zeleny sedimentation was measured according to AACC methods.

#### Soil health measurements

Soil organic carbon was measured by the Walkley-Black method.^23^ Microbial biomass carbon and nitrogen were measured by chloroform fumigation-extraction.^35^ Arbuscular mycorrhizal colonization was assessed by staining and microscopic examination.^36^

#### Greenhouse gas emissions

Net greenhouse gas emissions were calculated using IPCC methodology,^37^ including emissions from fertilizer production and application, irrigation pumping, and diesel combustion. Global warming potentials used were 298 for N_2_O and 1 for CO_2_.

#### Energy balance

Energy input included diesel, fertilizers, pesticides, seeds, and machinery. Energy output was calculated from grain and straw yields using standard energy coefficients.^18^

#### Economic analysis

Partial economic budget was calculated using local prices for inputs and outputs. Costs included fertilizers, pesticides, seeds, irrigation, and AI service. Revenue included grain value and protein premium.

### Quantification and statistical analysis

All statistical analyses were performed using R software version 4.3.2.^19^ Data were checked for normality using the Shapiro-Wilk test and for homogeneity of variance using Levene’s test. Paired t-tests were used to compare traditional and AI treatments for all measured variables. Effect sizes were calculated as Cohen’s d. Statistical significance was set at *p* < 0.05. Data are presented as mean ± standard deviation (*n* = 4). In figures, asterisks indicate significant differences (∗*p* < 0.05, ∗∗*p* < 0.01, ∗∗∗*p* < 0.001) based on paired t-tests. Exact *n* values are provided in figure and table legends.

### Additional resources

Not applicable.
